# Whole plastid genome of *Anemone taipaiensis* (Ranunculaceae), an endemic herb plant in China

**DOI:** 10.1080/23802359.2019.1689864

**Published:** 2019-11-14

**Authors:** Cai-Xia Huang, Mi-Li Liu, Ting Yang, Xue-Yi Chen, Qi-Yuan Gao

**Affiliations:** a College of Water Conservancy and Hydropower Engineering, Gansu Agricultural University, Lanzhou, PR China;; b Key Laboratory of Resource Biology and Biotechnology in Western China, Ministry of Education, College of Life Sciences, Northwest University, Xi’an, PR China

**Keywords:** *Anemone taipaiensis*, phylogenetic relationship, plastid genome

## Abstract

*Anemone taipaiensis* W. T. Wang is an endemic herb species in Shaanxi province (China). Here, we first characterized its whole plastid genome *via* pair-end sequencing method. The whole chloroplast genome was 156,659 bp in size, including a large single-copy (LSC) region of 78,439 bp, a small single-copy (SSC) region of 16,178 bp, and two repeat regions (IRs) of 31,021 bp. A total of 135 genes, including 91 protein-coding genes, 36 tRNA, and 8 rRNA genes were identified in *A. taipaiensis*. The phylogenetic analysis showed that *A*. *taipaiensis* have a close relationship with congeneric species *A. trullifolia.*

*Anemone taipaiensis* W. T. Wang (Ranunculaceae) is an endemic herb species in Shaanxi Province of China, and the rhizomes of this plant have been used in traditional Chinese medicine for the treatment of rheumatism and phlebitis. In recent years, *A*. *taipaiensis* has been widely studied as a new anti-tumor glycoside (Wang et al. 2013). However, the molecular evolution of *A*. *taipaiensis* is still poorly understood. The plant chloroplast (cp) DNA provided valuable phylogenetic information, owning to its conserved genome structures and slowly evolutionary rates (Wu and Ge 2012). Herein, we characterized the whole plastid genome of *A*. *taipaiensis* based on the Illumina next generation sequencing technology.

The fresh leaves of *A*. *taipaiensis* from a single tissue material were sampled from Qingling Mountains in China (N34.1036°, E107.2950°). We extracted the high quality DNA of *A*. *taipaiensis* using a modified CTAB procedure (Doyle and Doyle 1987). We deposited the voucher specimen (No. ATLZH2015708) into the Northwest University Museum (NUM). Then, the high quality DNAs were subjected to Illumina sample preparation and pair-read sequencing was conducted on the Illumina Hiseq 2500 platform in Novogene Bioinformatics Technology Co., Ltd (Beijing, China). Reference-guided assembly method was used to reconstruct the chloroplast genome with the program MITObim version 1.7 (Hahn et al. 2013). In this process, we used *Anemone trullifolia* (NC_039456) as reference in order to obtain accurate sequence. The complete plastid genome was annotated using the online program Dual Organellar Genome Annotator (DOGMA, Wyman et al. 2004). Eventually, the whole cp genome sequence of *A*. *taipaiensis* has been submitted to GenBank with the accession number: MN080710. Circular plastid genome maps were drawn using OGDRAW1 (Lohse et al. 2013).

The complete genome of *A*. *taipaiensis* was a quadripartite circular DNA molecule with a length of 156,659 bp, which comprises a large single-copy (LSC) region of 78,439 bp and a small single-copy (SSC) region of 16,178 bp, separated by two inverted repeat regions (IRs) of 31,021 bp. The cp genome contains 135 genes, including 91 protein-coding genes, 36 tRNA, and 8 rRNA. A total of 14 genes (*atpF*, *ndhA*, *ndhB*, *petB*, *petD*, *rpl2*, *rpl16*, *rpoC1*, *trnA*-*UGC*, *trnG*-*UCC*, *trnI*-*GAU*, *trnK*-*UUU*, *trnL*-*UAA*, and *trnV*-*UAC*) contained one intron, and three genes (*clpP*, *ycf3*, and *rps12*) contained two introns. The overall GC content of *A*. *taipaiensis* cp genome is 37.7%, while the corresponding values of LSC, SSC, and IR regions are 35.7%, 31.4%, and 41.9%, respectively.

In order to investigate the phylogenetic position of *A*. *taipaiensis*, the plastid genomes of 17 species within Ranunculaceae were downloaded from NCBI. Eighteen sequences were aligned using MAFFT (Katoh and Standley 2013) in GENEIOUS R8 with the default parameters set. Phylogenetic analysis was performed using RAxML version 7.2.8 (Stamatakis 2006) with 1000 bootstrap replicates. The results showed that *A*. *taipaiensis* was placed as a sister to the congeneric *A*. *trullifolia* with high bootstrap value (Figure 1).

**Figure 1.**
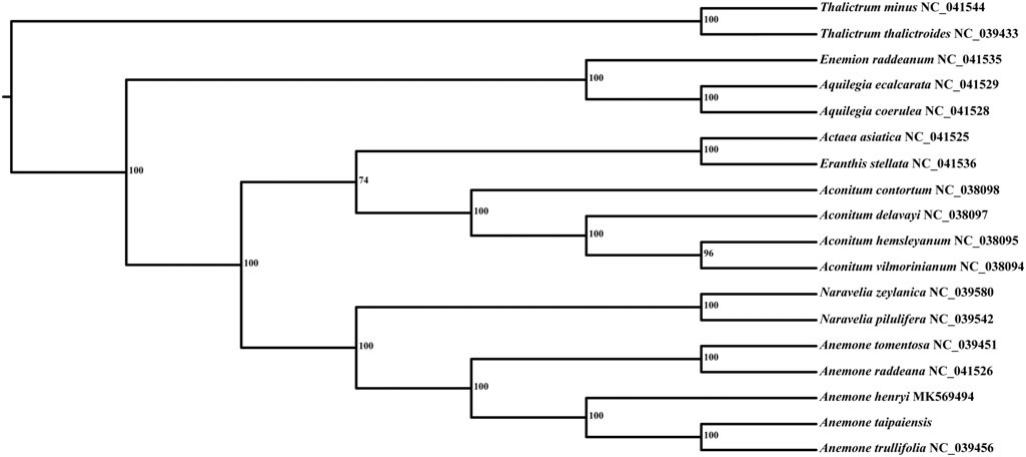
Phylogenetic relationship tree based on 18 plastid genome sequences.
